# HLA-A2 Promotes the Therapeutic Effect of Umbilical Cord Blood-Derived Mesenchymal Stem Cells in Hyperoxic Lung Injury

**DOI:** 10.3390/bioengineering9040177

**Published:** 2022-04-18

**Authors:** Jihye Kwak, Wankyu Choi, Yunkyung Bae, Miyeon Kim, Soojin Choi, Wonil Oh, Hyejin Jin

**Affiliations:** Biomedical Research Institute, MEDIPOST Co., Ltd., Gyeonggi-do 463-400, Korea; jihyek@medi-post.co.kr (J.K.); choiwk@medi-post.co.kr (W.C.); byk819@medi-post.co.kr (Y.B.); eldjfls3@medi-post.co.kr (M.K.); sjchoi@medi-post.co.kr (S.C.); wioh@medi-post.co.kr (W.O.)

**Keywords:** UCB-MSCs, surface maker array, HLA-A2, anti-inflammation, T cell suppression, hyperoxic lung damage, quality control

## Abstract

Mesenchymal stem cells (MSCs) are one of the most extensively studied stem cell types owing to their capacity for differentiation into multiple lineages as well as their ability to secrete regenerative factors and modulate immune functions. However, issues remain regarding their further application for cell therapy. Here, to demonstrate the superiority of the improvement of MSCs, we divided umbilical cord blood-derived MSCs (UCB-MSCs) from 15 donors into two groups based on efficacy and revealed donor-dependent variations in the anti-inflammatory effect of MSCs on macrophages as well as their immunoregulatory effect on T cells. Through surface marker analyses (242 antibodies), we found that HLA-A2 was positively related to the anti-inflammatory and immunoregulatory function of MSCs. Additionally, HLA-A2 mRNA silencing in MSCs attenuated their therapeutic effects in vitro; namely, the suppression of LPS-stimulated macrophages and phytohemagglutinin-stimulated T cells. Moreover, HLA-A2 silencing in MSCs significantly decreased their therapeutic effects in a rat model of hyperoxic lung damage. The present study provides novel insights into the quality control of donor-derived MSCs for the treatment of inflammatory conditions and diseases.

## 1. Introduction

Mesenchymal stem cells (MSCs) have a self-renewal capacity, are multipotent, and secrete various trophic factors [1,2]. Umbilical cord blood-derived MSCs (UCB-MSCs) are particularly advantageous as they are easily obtained, exhibit a high proliferation rate, express various beneficial paracrine factors, and are well-tolerated upon transplantation because of their low immunogenicity and high immunomodulatory ability [3,4].

MSCs have major potential in human regenerative medicine owing to their ability to migrate to areas of damage and suppress inflammation [5]. The early conjecture was that MSCs replaced the cells of the damaged area but it was subsequently shown that MSC-secreted factors support the regenerative process in damaged tissues, induce neovascularization, and regulate the immune system [6]. Currently, MSC research is focused on the secretory function as it is presumed that the paracrine effect of MSCs underlies their therapeutic efficacy. These characteristics have extended the scope of MSC clinical applications [7]. Human MSCs (hMSCs) have potential uses as a cell therapy for various conditions, including rheumatoid arthritis, Alzheimer’s disease, graft-versus-host disease, bronchial pulmonary disease (BPD), and chronic obstructive pulmonary disease [7,8,9]. 

Despite years of basic and preclinical research, the clinical outcomes of MSC use vary greatly and are often unsatisfactory [10,11]. The apparent gap between in vitro and clinical results may be due to the unsuccessful engraftment of MSCs at lesion areas, low cell viability within the recipient, and donor-dependent cellular characteristics [12,13]. MSCs are predicted to exert varying effects under different circumstances as they are live cells and are subject to donor variations [2,12,13,14]. According to a recent study, the heterogeneity of MSCs affects their proliferation, differentiation, and immunophenotype as well as their secretome [15], thus limiting the clinical application of MSCs despite their various advantages. Therefore, criteria for screening functionally potent stem cells in order to maximize their therapeutic efficacy should be established.

The mechanisms underlying the immunomodulatory effects of MSCs in various diseases remain unclear but are generally mediated via direct contact with immunomodulatory cell types as well as through the secretion of various immunomodulatory factors [16,17]. These MSC-derived secretory factors include TGF-β1, hepatocyte growth factor (HGF), prostaglandin E2 (PGE2), indoleamine 2,3-dioxygenase (IDO), and IL-10 [18,19]. MSC therapeutic effects are partly mediated via secretory protein-induced macrophage polarization, which leads to the suppression of inflammation [20]. In particular, M2 macrophage polarization drives the production of anti-inflammatory cytokines [20,21].

Various functional surface proteins are recognized as markers that indicate the biological features and functions of cells [22]. They are also utilized in the process of MSC isolation [22,23]. Despite their significance, surface proteins have not been extensively studied with regard to their direct association with MSC immunomodulatory and anti-inflammatory activities. According to a recent study, MSCs suppress T cell activation via the activity of surface marker CD200 [24,25]. However, CD200 alone cannot determine the therapeutic effects of MSCs, which highlights the need to identify additional marker proteins and investigate the signaling pathways through which these might promote immunosuppression.

In the present study, HLA-A2 marker expression was evaluated through surface protein identification in order to screen MSCs with a therapeutic potential in inflammatory conditions. In addition, the variation in the immunomodulatory and anti-inflammatory effects of UCB-MSCs was examined in vitro based on the expression of HLA-A2 as well as in animal models of BPD.

The current findings provide an insight into the anti-inflammatory mechanism of MSCs in inflammatory diseases in addition to establishing screening criteria for MSCs with a therapeutic potential.

## 2. Materials and Methods

### 2.1. Cell Culture and Characterization

UCB samples were collected from human umbilical veins isolated following neonatal delivery after obtaining informed maternal consent. This process was approved by the Institutional Review Board of MEDIPOST Co., Ltd. (MP-2015-6, Seongnam, Korea). UCB-MSCs, separated from mononuclear cells (MNCs) using Ficoll-Paque^TM^ PLUS (GE Healthcare, Uppsala, Sweden), were washed and cultured in minimum essential medium alpha (α-MEM; Gibco/Invitrogen, Carlsbad, CA, USA) supplemented with 10% fetal bovine serum (FBS; Gibco) at 37 °C in a 5% CO_2_ atmosphere [26]. UCB-MSCs from 15 donors were used in this study. Detailed information related to the UCB-MSCs is provided in Table S1. 

To test the cell surface marker expression, the cells were stained for 15 min at room temperature using fluorescein isothiocyanate (FITC)-conjugated antibodies against human CD14, CD34, and HLA-DR (BD Biosciences, Franklin Lakes, NJ, USA) as well as phycoerythrin (PE)-conjugated antibodies against human CD73, CD90, CD166, HLA-A2 (BD Biosciences), and CD105 (Invitrogen, Carlsbad, CA, USA). Isotype-matched mouse antibodies were used as the controls. The cells were washed with phosphate-buffered saline (Gibco) and fixed with 1% (*v*/*v*) paraformaldehyde (Sigma-Aldrich, St. Louis, MO, USA). The cells were analyzed via flow cytometry on a FACSCalibur instrument (BD Biosciences) and the percentage of cells expressing surface antigens was calculated for 10,000 gated-cell events. To evaluate the multilineage potential, the cells were incubated in specific media to induce their differentiation into osteocytes, chondrocytes, and adipocytes. After stimulation, the multilineage potential was analyzed, as previously reported [27]. Briefly, osteocyte formation was assessed by measuring the level of alkaline phosphatase (ALP) staining (Sigma-Aldrich, St. Louis, MO, USA), chondrocyte formation was evaluated based on safranin O staining (Sigma-Aldrich, St. Louis, MO, USA), and adipocyte formation was assessed based on the staining of accumulated lipid vacuoles using Oil Red O (Sigma-Aldrich, St. Louis, MO, USA).

### 2.2. Macrophage Assays

The mouse macrophage cell line RAW 264.7 was obtained from the American Type Culture Collection (Manassas, VA, USA). The cells were cultured at a density of 1 × 10^5^ cells/well in serum-free RPMI-1640 and stimulated with 1 µg/mL lipopolysaccharide (LPS; derived from *Escherichia coli* O55:B5; L6529; Sigma-Aldrich, St. Louis, MO, USA). The MSCs (1 × 10^5^ cells/well) were co-cultured with LPS-activated RAW 264.7 and the supernatants were collected after 48 h. The supernatant was collected and clarified by centrifugation at 1200 rpm for 5 min. The mouse TNF-α levels in the supernatant were measured via an enzyme-linked immunosorbent assay (ELISA; R&D Systems, Minneapolis, MN, USA) [28]. 

### 2.3. Mixed Lymphocyte Reaction (MLR) Assay

Prior to MLR processing, stimulated PBMCs and UCB-MSCs were inactivated via a treatment with 10 μg/mL mitomycin-C (Sigma-Aldrich, St. Louis, MO, USA) for 1 h at 37 °C. The UCB-MSCs (1 × 10^3^ cells/well) were seeded and maintained at 37 °C in a humidified incubator for 4 h and then co-cultured with PBMCs (1 × 10^5^ cells/well; Allcells, Boston, MA, USA) from different donors. Phytohemagglutinin (PHA) (5 μg/mL, Roche)-treated PBMCs were used as the positive control. After a co-culture with the MSCs, the PBMCs were maintained for 5 days in an RPMI-1640 medium (Gibco) supplemented with 10% FBS and gentamycin. The proliferation of PBMCs was measured using a cell proliferation BrdU (colorimetric) ELISA kit (Roche). The supernatants were then collected to measure the levels of the immunoregulatory cytokine PGE_2_ using an ELISA kit (R&D Systems, Inc., Minneapolis, MN, USA).

### 2.4. Flow Cytometry and Cell Surface Antibody Screening

To screen the surface markers of hMSCs, 242 antibodies (Figure S1) were lyophilized in 96-well plates (BD Lyoplates; BD Biosciences) at 0.5 µg/well and incubated with 500,000 MSCs per well. After 20 min of reconstitution on ice, the washed cells were stained with an Alexa Fluor^®^ 647-conjugated goat anti-mouse IgG secondary antibody (Molecular Probes, Eugene, OR, USA). Flow cytometry was performed to confirm the surface marker expression on a FACSCalibur instrument (BD Biosciences). Flow cytometry data were analyzed using Excel 2013 (Microsoft, Redmond, WA, USA) to generate heat maps [28].

### 2.5. Small Interfering RNA-Mediated Knockdown

siRNA against HLA-A2 or control siRNA was purchased from Dharmacon (Chicago, IL, USA). siRNA was transfected into cells using the Dharmafect Reagent (Dharmacon) according to the manufacturer’s instructions. Naïve cells were cultured in a basic medium without transfection. The siRNA pools consisted of four different siRNA duplexes (Table S2). To confirm the knockdown efficiency, qPCR was performed using a LightCycler 480 (Roche, Mannheim, Germany). TaqMan probes were designed with the Universal Probe Library Assay Design Center and used to quantitatively detect the mRNA transcript levels of the genes encoding the HLA-A2 and β-actin. The relative expression levels of the genes of interest were calculated using the comparative threshold cycle method (2^−^^ΔΔCt^) and normalized to the β-actin mRNA expression.

### 2.6. Hyperoxic Lung Injury in Vivo Model

All animal experiments were reviewed and approved by the Institutional Animal Care and Use Committee of MEDIPOST Co., Ltd. (MP-LAR-2016-7-1, Seongnam, Korea). This study was also performed in accordance with the institutional and National Institutes of Health guidelines for laboratory animal care. Rat pups (10 g) were delivered by pregnant Sprague Dawley rats (Samtako Bio Korea Co. Ltd., Osan, Korea). The experimental designs are shown in Figure S2 and Table S3. Within 10 h after birth, the rat pups were randomly assigned to the following five groups: (i) normal; (ii) BPD; (iii) BPD + naïve MSCs; (iv) BPD + control siRNA MSCs; or (v) BPD + HLA-A2 siRNA MSCs. We evaluated the HLA-A2 expression before the MSC injection (Figure S2). The control group rats were kept under normoxic conditions whereas the rats of the hyperoxic groups were kept in hyperoxic chambers under 90% oxygen from birth to postnatal day 14, as previously reported. To avoid oxygen toxicity, nursing mother rats were rotated daily between litters maintained under normoxic and hyperoxic conditions. The MSCs (1 × 10^5^/head, P6) were washed with saline after washing twice with pre-warmed MEM-α without phenol red. After the saline washing, the MSC suspensions were prepared in saline and injected intratracheally at P5, as previously described [29]. The survival rates and health condition of all rat pups were monitored daily. The rat pups were anesthetized via an intraperitoneal injection of pentobarbital (60 mg/kg) at P14. Eleven to fifteen animals were assigned to each group. Whole lung tissues were obtained from sacrificed rat pups and fixed with 4% paraformaldehyde. Fixed lung tissues were embedded in paraffin, sectioned, and stained with hematoxylin and eosin (H&E). MLI was used to measure the level of alveolarization by dividing the total length of the lines drawn across the lung section by the number of intercepts encountered, as reported previously [29]. Randomly selected sections (>3) per rat and 100 fields per section were analyzed. To confirm the transplantation of the injected MSCs into the lung tissues, CD11b/c or CD163 were stained with primary antibodies, followed by detection with Alexa Fluor^®^ 488—or Cy3-conjugated secondary antibodies (Jackson ImmunoResearch Europe Ltd., Newmarket, UK). The nuclei in the lung tissues were counterstained with Hoechst 33342. The stained lung tissues were imaged and analyzed using an LSM 800 confocal microscope. The concentrations of rat TNF-α and IL-10 in the BALF samples were determined using an ELISA, as previously described. 

### 2.7. Statistical Analysis

All data were presented as the mean ± standard deviation (SD) of the values obtained in experiments performed at least in triplicate. The statistical analysis was performed using a one-way analysis of variance, followed by a least significant difference (LSD) post hoc test in Prism 6 software (GraphPad, San Diego, CA, USA). The statistical significance was set at *p* < 0.05.

## 3. Results

### 3.1. Potential Efficacy of UCB-MSCs in Vitro

**Figure 1:**
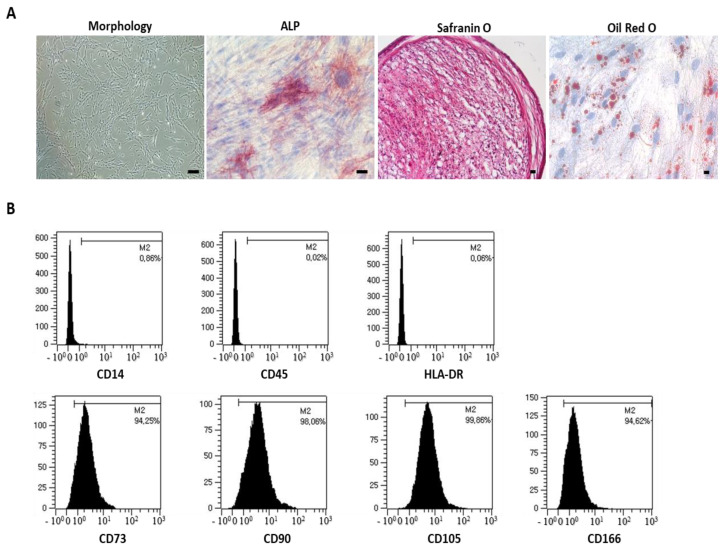
Characterization of MSCs at P6. (**A**) Morphological analysis shows adherent spindle-shaped cells. During incubation in a specialized induction medium, multilineage potential was demonstrated by staining typical multilineage markers. Osteogenic cells were analyzed based on ALP levels. Chondrogenic cells accumulated sulfated proteoglycans stained with Safranin O. Adipogenic cells exhibited increased lipid vacuoles within the cytoplasm via Oil Red O staining. Nuclei were counterstained with hematoxylin. Scale bar: 100 µm. (**B**) Flow cytometric analysis of cells based on the cell surface expression of typical MSC markers. Cells were negative for CD14, CD45, and HLA-DR but strongly positive for CD73, CD90, CD105, and CD166. M2-related expression was noted.

At P6, the MSC characteristics were identified and their spindle shape was observed. A multipotent differentiation into osteocytes, adipocytes, and chondrocytes was determined based on ALP, Oil Red O, and Safranin O staining. The immunophenotype results confirmed the negative expression of CD14, CD45, and HLA-DR as well as the positive expression of CD73, CD90, CD105, and CD166 (Figure 1) [30].

**Figure 2:**
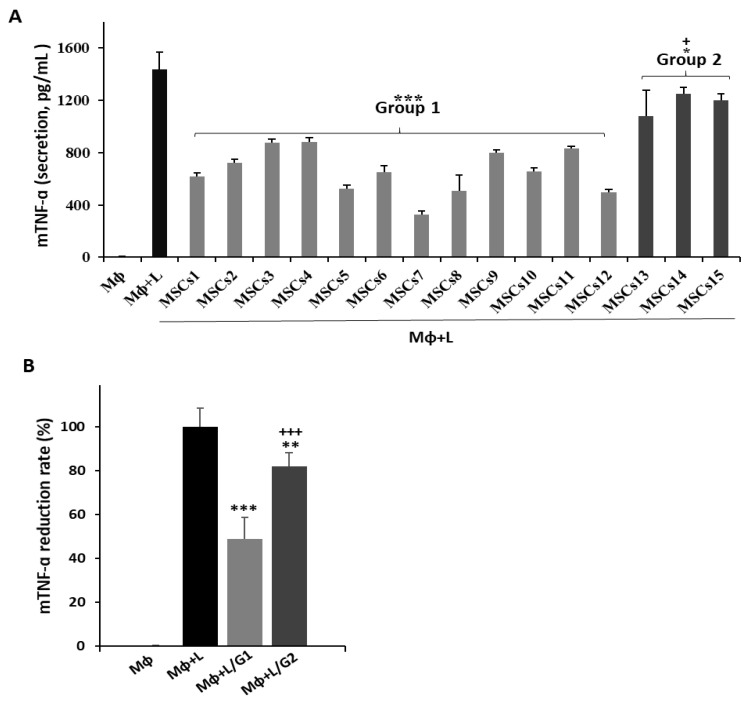
Anti-inflammatory effect of UCB-MSCs in LPS-stimulated macrophages. RAW 264.7 cells were exposed to LPS and co-cultured with UCB-MSCs for 2 days. (**A**,**B**) Cell supernatants were analyzed for inflammatory cytokine TNF-α levels via ELISA. Fifteen different MSC lots were evaluated, all of which exhibited anti-inflammatory effects. MSCs were divided into two groups based on the extent of TNF-α suppression; namely, Group 1 (MSCs1 to MSCs12) and Group 2 (MSCs13 to MSCs15). (**A**) Error bars represent the means ± SD, *n* = 5 per group; *** *p* < 0.001, ** *p* < 0.01, * *p* < 0.05 vs. MΦ + L, +++ *p* < 0.001, + *p* < 0.05 vs. Group 1. (**B**) Data are presented as the mean ± SD for *n* = 5 (MΦ, MΦ + L), *n* = 60 (MΦ + L/G1), or *n* = 12 (MΦ + L/G2) per group; *** *p* < 0.001 vs. MΦ + L, + *p* < 0.05 vs. MΦ + L/G1. MΦ: macrophage; L: LPS; G1: Group 1; G2: Group 2.

**Figure 3:**
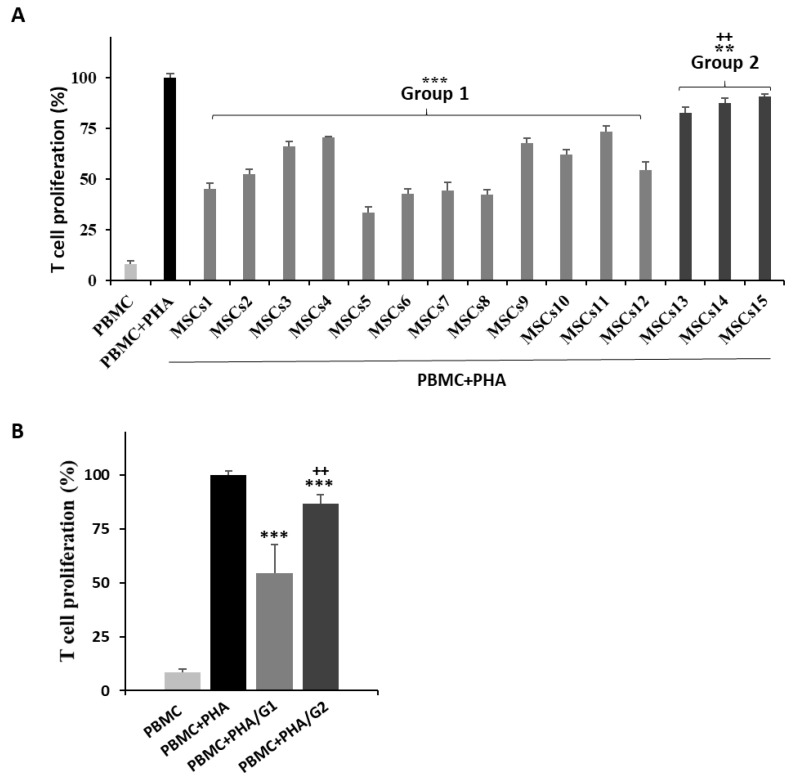
Effect of UCB-MSCs on human T cell proliferation in the MLR assay. PHA-stimulated PBMCs were co-cultured with MSCs for 3 days. (**A**,**B**) The proliferation of T cells is shown as a percentage relative to the positive control (PBMC + PHA; set to 100%). Fifteen different MSCs were evaluated to confirm their T cell-suppressive effects. All MSCs suppressed T cell proliferation. Based on MLR assay results, MSCs were classified two groups depending on the extent of T cell suppression; namely, Group 1 (MSCs1 to MSCs12) and Group 2 (MSCs13 to MSCs15). (**A**) Error bars represent means ± SD, *n* = 5 per group; *** *p* < 0.001, ** *p* < 0.01 vs. PBMC + PHA, ++ *p* < 0.01 vs. Group 1. (**B**) Data are presented as the mean ± SD for *n* = 5 (PBMC, PBMC + PHA), *n* = 60 (PBMC + PHA/G1), or *n* = 12 (PBMC + PHA/G2) per group; *** *p* < 0.001 vs. PBMC + PHA, ++ *p* < 0.01 vs. PBMC + PHA/G1. PBMC: peripheral blood mononuclear cell; PHA: phytohemagglutinin; G1: Group 1; G2: Group 2.

Two experiments were conducted on 15 MSC lots from different donors in order to verify the lot variation in the immunoregulatory potential of MSCs. To evaluate the anti-inflammatory effects of the MSCs, RAW 264.7 macrophages were stimulated with LPS to induce M1 polarization and a co-culture with the MSCs was performed. The levels of inflammatory cytokine TNF-α in the supernatant were then analyzed. All 15 MSC lots induced a decrease in TNF-α secretion and the rate of reduction varied between the lots. The MSCs were divided into two groups based on the significance of TNF-α suppression (Figure 2A). Group 1 included 12 MSC lots, which induced a ≥ 35% reduction of the TNF-α levels during the co-culture; Group 2 contained three lots with a significantly lower rate of TNF-α suppression than those in Group 1 (Figure 2B). Second, to monitor the level of immunosuppression, the PBMCs were stimulated with PHA and the level of suppression under the co-culture with MSCs was assessed via an MLR analysis. All 15 MSC lots suppressed T cell proliferation and the rate of reduction varied across the different lots. The MSCs were divided into two groups based on the statistical significance (Figure 3B). Group 1 included 12 MSC lots with ≥25% T cell suppression and Group 2 included three lots with significantly lower suppressive effects (Figure 3B). Both experiments confirmed that the MSC lots had a varying immunosuppressive capacity, highlighting the need for potent MSC screening criteria.

### 3.2. Identification of UCB-MSC Surface Markers Based on Efficacy

Cell surface markers (proteins, lipids, glycosylation, etc.) can be used to distinguish unique cell subpopulations. In MSC research, the surface marker expression is key for inferring the cellular function and thus these serve as reliable indicators for cell screening [31].

**Figure 4:**
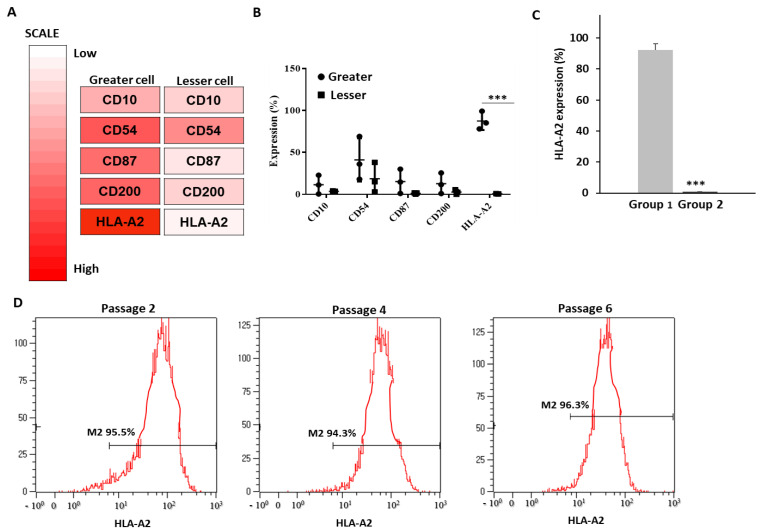
Screening for UCB-MSC cell surface markers. We prepared three lots from each group, labeling those from Group 1 as greater cells and those from Group 2 as lesser cells. (**A**) Heat map analysis showing cell surface markers upregulated in greater compared with lesser cells. (**B**) To confirm the increase in surface protein expression observed during screening, the CD10, CD54, CD200, and HLA-A2 expression was assessed via flow cytometry (means ± SD, *n* = 3; *** *p* < 0.01). HLA-A2 expression was lowest in lesser cells. (**C**) To confirm the downregulation of cell surface proteins shown in panel B, HLA-A2 expression on UCB-MSCs from 15 different donors (Group 1 and Group 2) was analyzed via flow cytometry. HLA-A2 expression was significantly higher in Group 1 compared with Group 2. Data are presented as mean ± SD for *n* = 36 (Group 1) or *n* = 9 (Group 2) per group. *** *p* < 0.001. (**D**) During expansion, HLA-A2 expression was measured via flow cytometry at the indicated passages. Positive M2-related expression was noted.

To examine the differences in the surface marker expression between the two groups shown in Figure 2 and Figure 3, three lots from each group were selected and a cell surface array of 242 proteins was performed. Five primary candidates (CD10, CD54, CD87, CD200, and HLA-A2) were selected based on the differences in expression between Group 1 (greater cell) and Group 2 (lesser cell), with the results indicating higher expression levels for all five markers in Group 1 (Figure 4A). The most significant difference in expression was observed for the HLA-A2 protein (Figure 4B). The other markers did not exhibit significant differences in expression due to lot variations. In addition, only Group 1 cells (12 lots) had an average expression of ≥94% whereas the expression was <1% in all Group 2 cells (3 lots). To gain a further insight, HLA typing was performed; the results were similar to those of flow cytometry (data not shown). This indicated that HLA-A2 was a robust marker of cell efficacy, suitable for a rapid assessment via flow cytometry. In addition, an analysis of two BM-MSC lots revealed that one lot had a negative (0.9%) and the other had a positive (90.5%) HLA-A2 expression, indicating that the HLA-A2 expression also varied among the MSCs from other sources. We conducted the same macrophage and PBMC experiments using BM-MSCs. The MSCs under both lots induced a significant reduction in TNF-α levels and T cell proliferation. However, the efficacy of the BM-MSC1 cells was significantly lower compared with that of the BM-MSC2 cells (Figure S4). Moreover, there was no change in the HLA-A2 expression during the passaging from P2 to P6. Taken together, these results suggested that the cells positive for HLA-A2 expression exerted potent anti-inflammatory and immunomodulatory effects whereas those with a negative HLA-A2 expression exhibited poor efficacy, indicating that HLA-A2 may be a candidate marker for MSC selection.

### 3.3. Knockdown of HLA-A2 in UCB-MSCs Compromises Their Therapeutic Effects in Vitro

**Figure 5:**
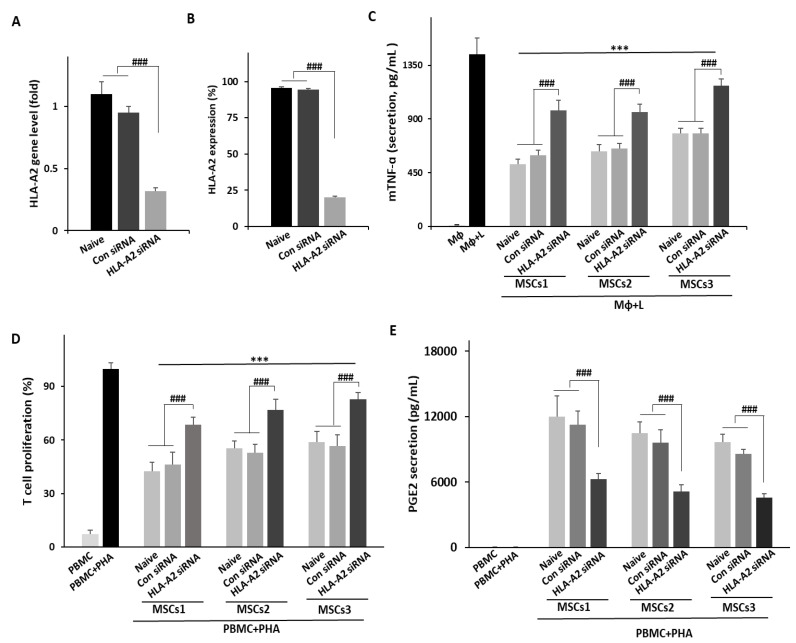
HLA-A2 knockdown in MSCs attenuates their therapeutic effects in vitro. Cells were transfected with control siRNA or HLA-A2 siRNA. (**A**) Gene expression and (**B**) protein levels of HLA-A2 were compared relative to those in non-transfected MSCs (naïve). (**A**,**B**) Error bars represent means ± SD, *n* = 3 per group; ### *p* < 0.001. (**C**,**E**) MSCs from three different lots of Group 1 were evaluated to confirm their therapeutic effects in vitro. (**C**) HLA-A2 siRNA-treated MSCs were co-cultured with LPS-induced RAW 246.7 cells and TNF-α levels were assessed via ELISA. Lower levels of TNF-α were detected in media from HLA-A2 knockdown MSCs. Error bars represent means ± SD, *n* = 3 per group; ### *p* < 0.001, *** *p* < 0.001 vs. MΦ + L. (**D**) Proliferation of human T cells based on MLR assays. PBMCs were co-cultured with MSCs. The proliferation of responding cells is shown as a percentage relative to the positive control (PBMC + PHA; set to 100%). (**E**) Levels of PGE2 in the culture media of cells from the MLR assay. Lower levels of PGE2 were noted in media from HLA-A siRNA MSCs. (**D**,**E**) Error bars represent means ± SD, *n* = 3 per group; ### *p* < 0.001, *** *p* < 0.001 vs. PBMC + PHA. MΦ: macrophage; L: LPS; PBMC: peripheral blood mononuclear cell; PHA: phytohemagglutinin.

To verify whether HLA-A2 was an optimal marker of MSC efficacy, three lots from Group 1 were transfected with siRNA to knock down the HLA-A2 expression. For each lot, three conditions were applied (naïve, con siRNA, and HLA-A2 siRNA). No significant difference was observed in the expression of typical MSC markers (Table S4). In addition, upon siRNA treatment, the HLA-A2 expression was significantly inhibited at the mRNA and protein levels (Figure 5A,B). We then repeated the macrophage and PBMC experiments in order to assess the immunoregulatory function of the transfected MSCs. HLA-A2 knockdown MSCs from all three lots induced a significant decrease in TNF-α levels. However, the rate of reduction was significantly lower in the HLA-A2 siRNA group than in the control groups (naïve or con siRNA), indicating compromised anti-inflammatory effects (Figure 5C). Identical changes in efficacy were observed in the PBMC experiments (Figure 5D). Therefore, the PGE2 levels in the supernatant were assessed and a significantly lower PGE2 secretion was noted in the HLA-A2 siRNA group than in the control group (naïve or con siRNA) for all three lots (Figure 5E). Taken together, these in vitro results suggested that HLA-A2 plays a critical role in the immunosuppressive effects of MSCs.

### 3.4. HLA-A2 Is Required for the Therapeutic Effects of UCB-MSCs in a Rat Model of Hyperoxic Lung Injury

**Figure 6:**
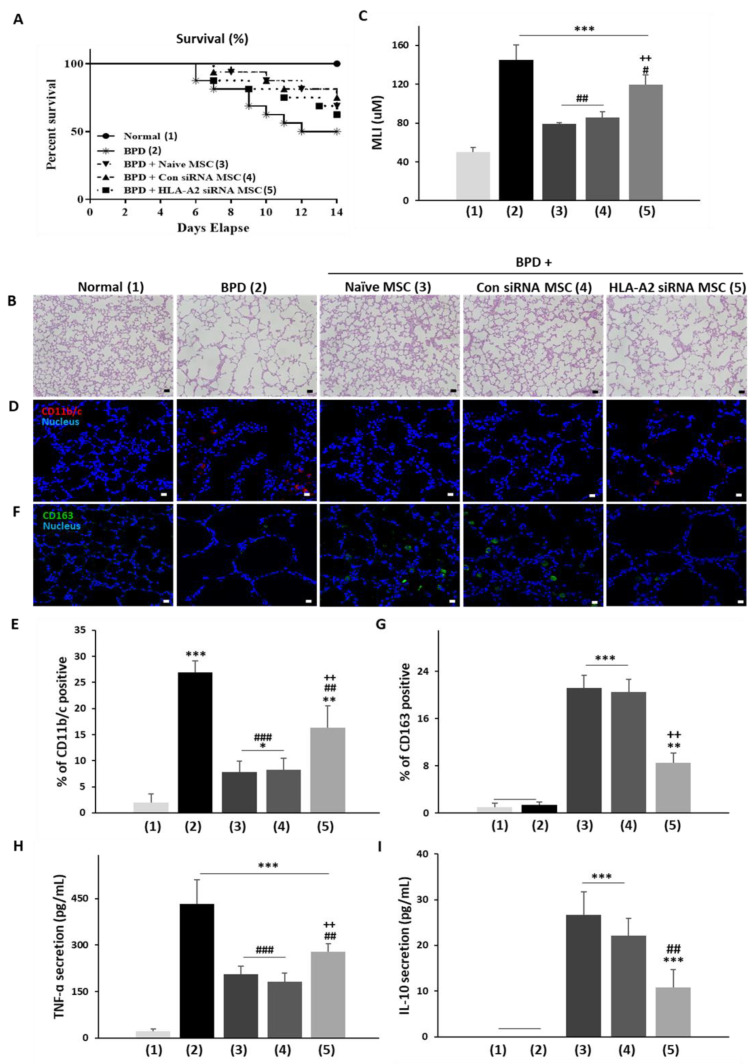
HLA-A2 knockdown suppressed the therapeutic efficacy of MSCs by inhibiting macrophage polarization. HLA-A2 siRNA-treated MSCs were intratracheally injected in BPD rats. (**A**) HLA-A2 siRNA-transfected MSCs were intratracheally injected on Day 5. Daily survival rates during a 14-day period from birth are presented as Kaplan–Meier survival curves. Lung tissues obtained on Day 14 were compared with those from the control group (Normal). (**B**) The mean linear intercept (MLI) values were evaluated by the degree of alveolarization. (**C**) Lung tissues were sectioned and stained with H&E. Results are presented as the mean ± SD for *n* = 11 (Normal), *n* = 15 (BPD, BPD with naïve MSC), or *n* = 14 (con siRNA MSCs, HLA-A2 siRNA MSCs) per group. (**D**,**F**) Immunofluorescence analysis for (**D**) CD11b/c or (**F**) CD163 in lung tissues. (**D**) Nuclei were stained with Hoechst 33342 (blue). Red (CD11b) and green (CD163) staining indicates positive cells. Scale bar: 10 µm. Expression of (**E**) CD11b/c and CD163 (**F**) was assessed as the percentage of positively stained cells. The levels of **(H**) TNF-α and (i) IL-10 in the BALF were analyzed by ELISA on Day 14. (**C**,**E**–**I**) Data are presented as the mean ± SD, *n* = 3 per group. *** *p* < 0.001, ** *p* < 0.01, * *p* < 0.05 vs. normal, ### *p* < 0.001, ## *p* < 0.001, # *p* < 0.05 vs. BPD, # *p* < 0.05 vs. BPD, ++ *p* < 0.01 vs control group (naïve MSCs or con siRNA MSCs). BPD: bronchopulmonary dysplasia; BLAF: bronchoalveolar lavage fluid.

BPD is a chronic lung disease caused by lung damage due to aberrant inflammation during the treatment of neonatal respiratory distress using a respirator [32]. Inflammation-induced lung damage then leads to an abnormal lung structure or incomplete lung development, which are characteristics of BPD [33]. Although it is the major cause of premature death and complications, no definite treatment or approved drugs have been established. The therapeutic effects of MSCs in BPD have been reported [29,34,35] with clinical trials currently underway. Using a hyperoxic lung injury rat model of BPD, naïve, con siRNA, and HLA-A2 siRNA-transfected MSCs were prepared and their HLA-A2 expression was measured. After the administration of MSCs via the airway, lung tissue inflammation and regeneration in premature rat pups were examined. Survival up to Day 9 after administration or Day 14 after birth was also determined. No deaths were observed in the normoxia group whereas pups in the hyperoxia group showed a 50% survival rate. Pups receiving naïve and con siRNA MSCs with HLA-A2 expression showed 70% and 75% survival rates, respectively, whereas the HLA-A2 knockdown group showed a 65% survival rate, indicating a lower rate of mortality than the control group (Figure 6A). An analysis of the alveolar size (MLI) revealed that the alveolar distance was higher in the hyperoxia group due to lung tissue damage whereas the alveolar size was significantly reduced in the control group of the MSCs with HLA-A2 expression (naïve or con siRNA), similar to that in the normoxia group. In contrast, the HLA-A2 siRNA group showed a significantly lower MLI than the control group (Figure 6B,C). To examine the anti-inflammatory efficacy based on the macrophage polarization, staining of the lung tissue was performed for inflammatory M1 marker CD11b and anti-inflammatory M2 marker CD163. Compared with the hypoxia group, the control MSC administration groups showed a significantly lower CD11b expression and a significantly higher CD163 expression. The HLA-A2 suppression group exhibited a significant change in the expression levels of CD11 and CD163 compared with the MSC control group (Figure 6D–G). In addition, the levels of inflammatory (TNF-α) and anti-inflammatory (IL-10) cytokines were measured in the BALF samples. The MSC control group exhibited a significant decrease in TNF-α levels and an increase in IL-10 levels compared with the hypoxia group. In contrast, the HLA-A2 siRNA group exhibited significantly lower rates of TNF-α suppression and an increase in IL-10 levels compared with the control group (Figure 6H,I). Based on these results, HLA-A2 was identified as an essential factor for the therapeutic effects of MSCs in a representative animal model of inflammatory disease, highlighting its value as a marker for MSC screening.

## 4. Discussion

MSCs are characterized by their adhesive properties and tri-lineage differentiation potential. The establishment of MSC screening criteria is an essential step in the standardization of stem cell research and preparation. The present study verified the surface marker criteria for efficacy in UCB-MSCs from 15 different donors. The UCB-MSCs used in this study were cultured in compliance with the Good Manufacturing Practice (GMP); these were adhered to a plastic surface and the conventional surface markers CD73, CD90, and CD105 were uniformly expressed but not CD14, CD45, and HLA-DR. These are general UCB-MSC markers that do not distinguish MSCs based on potential therapeutic efficacy. Thus, there is a need for the identification of specific markers indicative of a clinical performance of MSCs.

Beyond their value for cell characterization, surface proteins play an important role in intercellular contact, extracellular substrate interaction, cell signaling, and molecular transport in the cytoplasm [22,29]. The study of cell surface proteins through flow cytometry is extensively used in clinical settings.

In this study, a BD Lyoplate panel was used to assess the expression of 242 surface markers on the MSCs. A marker analysis focused on cell proliferation and aging based on our preliminary findings and those of previous studies. For instance, the level of vascular cell adhesion molecule-1 (CD106) was substantially reduced in bone marrow-derived MSCs after aging [36] in parallel with a decrease in the level of homing activity whereas the level of melanoma cell adhesion molecule CD146 was reduced due to replicative senescence along with a decrease in MSC differentiation and stemness, indicating the association between the marker suppression and ageing [37]. However, there is a general lack of studies on the anti-inflammatory and immunomodulatory effects of these panel markers.

To elucidate the underlying therapeutic mechanism of MSCs, we explored their anti-inflammatory effect in LPS-stimulated macrophages as well as their inhibitory effect on T cell proliferation.

Fifteen lots of UCB-MSCs from different donors with verified stem cell characteristics were tested for immunoregulatory activities and divided into two groups (Group 1: high efficacy and Group 2: low efficacy). A cell surface array analysis revealed the upregulation of five cell markers in Group 1 cells relative to those of Group 2; namely, CD10, CD54, CD87, CD200, and HLA-A2. Among them, the HLA-A2 expression was significantly different (≥90% expression in all three lots of Group 1 and <1% expression in Group 2 cells). Therefore, HLA-A2 was selected as a potential screening marker for a high MSC therapeutic efficacy. Furthermore, the observed pattern of the HLA-A2 expression was confirmed in all 15 UCB-MSC lots.

We then performed an HLA-A2 knockdown in UCB-MSCs and repeated the macrophage co-culture experiments to verify the correlation between the HLA-A2 expression in UCB-MSCs and their anti-inflammatory function. Analogously, we repeated the PBMC experiments to assess immunosuppression. The HLA-A2 knockdown compromised the MSC-induced suppression of TNF-α and T cell proliferation in the respective experiments. Moreover, the secretion of anti-inflammatory mediator PGE2 was also suppressed under the HLA-A2 knockdown. These findings supported the value of HLA-A2 as a marker with a functional involvement in the MSC-induced macrophage and T cell suppression.

To further verify the potential of HLA-A2 as a screening marker, we employed rats with hyperoxic lung injury as models of BPD, a representative inflammatory disease in which the therapeutic effects of MSCs have been verified. The therapeutic effects were compromised in rats treated with HLA-A2-deficient UCB-MSCs. Taken together, our in vivo experiment confirmed the functional role of HLA-A2 in the therapeutic effects of UCB-MSCs as well as its value for MSC screening. 

The human leukocyte antigen (HLA) in humans and H-2 in mice are respective orthologues of the major histocompatibility complex (MHC) [38]. MHCs include three classes of molecules; classes I, II, and III. MHC genes are found on the short arm of chromosome 6 [39,40]. MHC class I molecules are present on the surfaces of all somatic cells. All somatic cells display a “flag” in the form of MHC class I molecules to prove that they belong to a single individual [39,40]. MHC class I molecules are composed of α and β chains. The α chain determines the diversity of class I molecules, its identifier being the complex of HLA-A, HLA-B, and HLA-C, each of which is encoded by a respective eponymous gene [38]. The β chain forms the β2-microglobulin peptide with a molecular weight of 11,000 Da [41]. The α and β chains together or the α chain alone may form MHC class I molecules [41]. 

MSC-induced immunosuppression is known to involve the paracrine effects of secretory PGE2 and IL-10 on the immune cells. In addition to the immunomodulatory proteins secreted by MSCs [42], the HLA-G protein has been suggested to be a key immunoregulatory molecule [43,44]. Weng et al. reported that when a soluble divalent HLA-A2/IgG molecule (HLA-A2 dimer) was produced and loaded with the Tyr368-376 peptide, the Tyr/HLA-A2 dimer effectively suppressed T cell-mediated allo-responses in an in vitro experiment [45]. Another study reported a high level of expression of HLA-A2 in MSCs [46,47] although no significant correlation was found between the macrophage infiltration and HLA-A2 positivity in patients with a uveal melanoma [48]. Hence, no study has previously identified the role of HLA-A2 in MSC immunomodulation. Further studies are required to elucidate the specific mechanisms through which HLA-A2 promotes MSC-mediated immunoregulation.

## 5. Conclusions

In summary, the therapeutic efficacy of UCB-MSCs against neonatal hyperoxic lung injuries are mainly controlled by their anti-inflammatory effects. We provided evidence for the value of HLA-A2 as a marker for the screening of highly efficient MSCs for the treatment of inflammatory conditions.

## Data Availability

The data presented in this study are available on request from the corresponding author.

## Supplementary Materials

The following are available online at https://www.mdpi.com/article/10.3390/bioengineering9040177/s1, Figure S1: Cell surface antibody screening with Lyoplates; Figure S2: Experimental scheme of MSC administration in a hyperoxic rat model; Figure S3: Silencing of HLA-A2 expression; Figure S4: HLA-A2 expression in BM-MSCs from two different donors; Table S1: Basic information of UCB-MSCs; Table S2: Sequences of primers and siRNAs; Table S3: Groups for the in vivo experiment; Table S4: Surface marker expression among different stem cell populations.
