# Supplementary material for: HLA-A2 Promotes the Therapeutic Effect of Umbilical Cord Blood-Derived Mesenchymal Stem Cells in Hyperoxic Lung Injury
Source: Bioengineering (Basel). 2022 Apr 18;9(4):177. doi: 10.3390/bioengineering9040177 (PMC9029550; doi:10.3390/bioengineering9040177)
Supplement: Supplementary file 1 [file bioengineering-09-00177-s001.zip › bioengineering-1609284-supplementary.pdf]

Supplementary Materials:

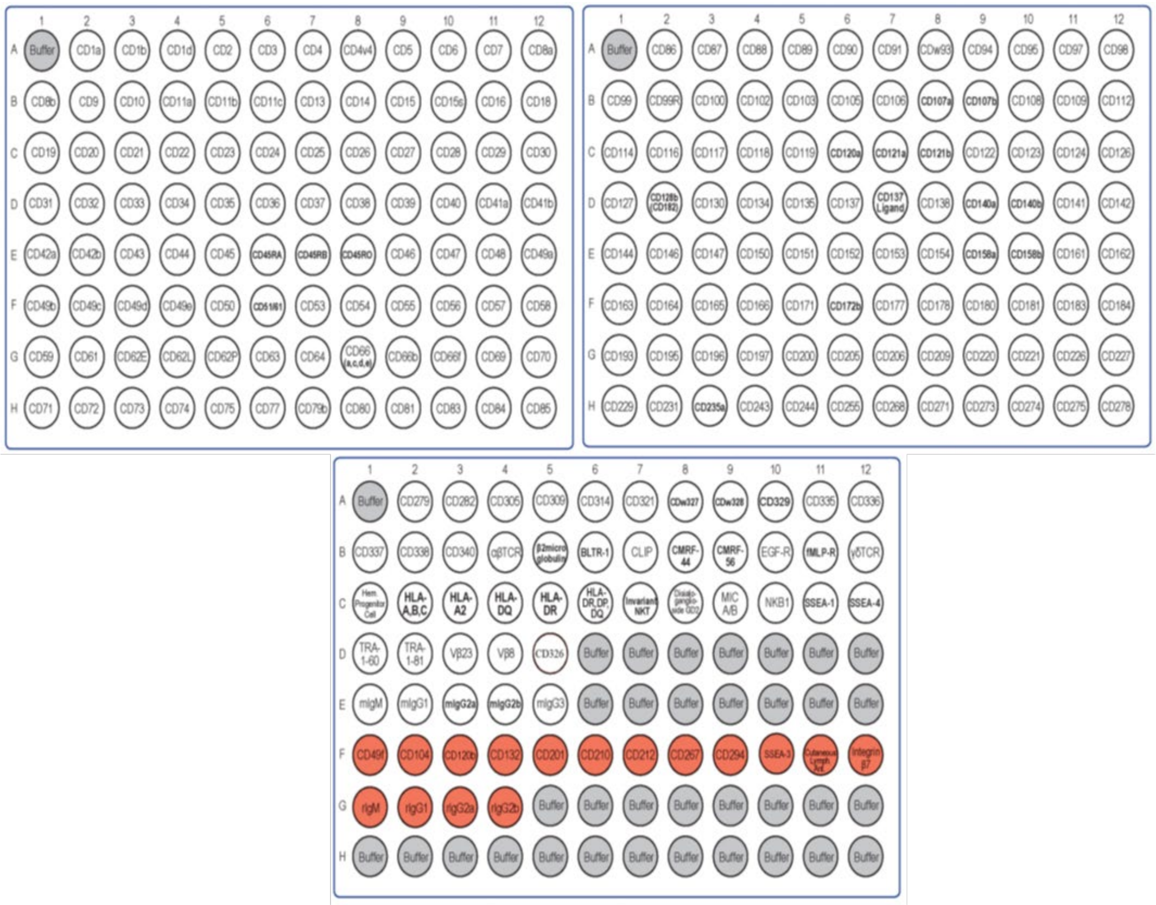

Figure S1. Cell surface antibody screening with Lyoplates. Lyophilized antibodies against 242 human cell surface markers are contained within the 96 well plates.

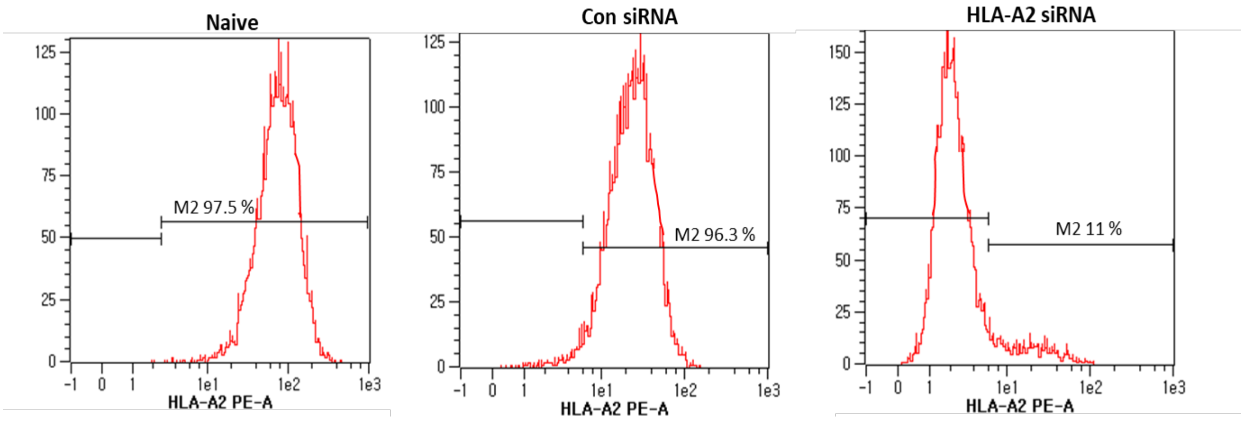

Figure S2. Silencing of HLA-A2 expression. UCB-MSCs were transfected with scramble siRNA (Con siRNAMSC) or HLA-A2 siRNA at P6 before injection in BPD model rats. Downregulated expression of HLA-A2 was confirmed via flow cytometry, confirming M2-related expression.

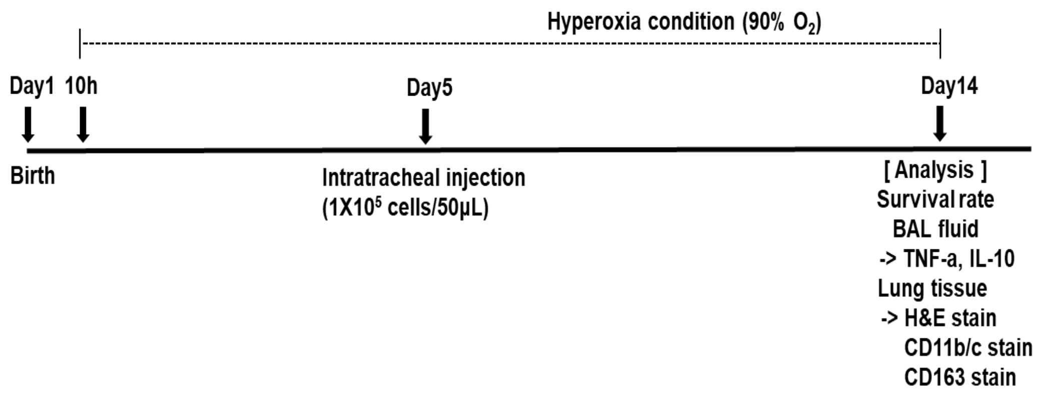

Figure S3. Experimental scheme of MSCs administration in the hyperoxic rat model.

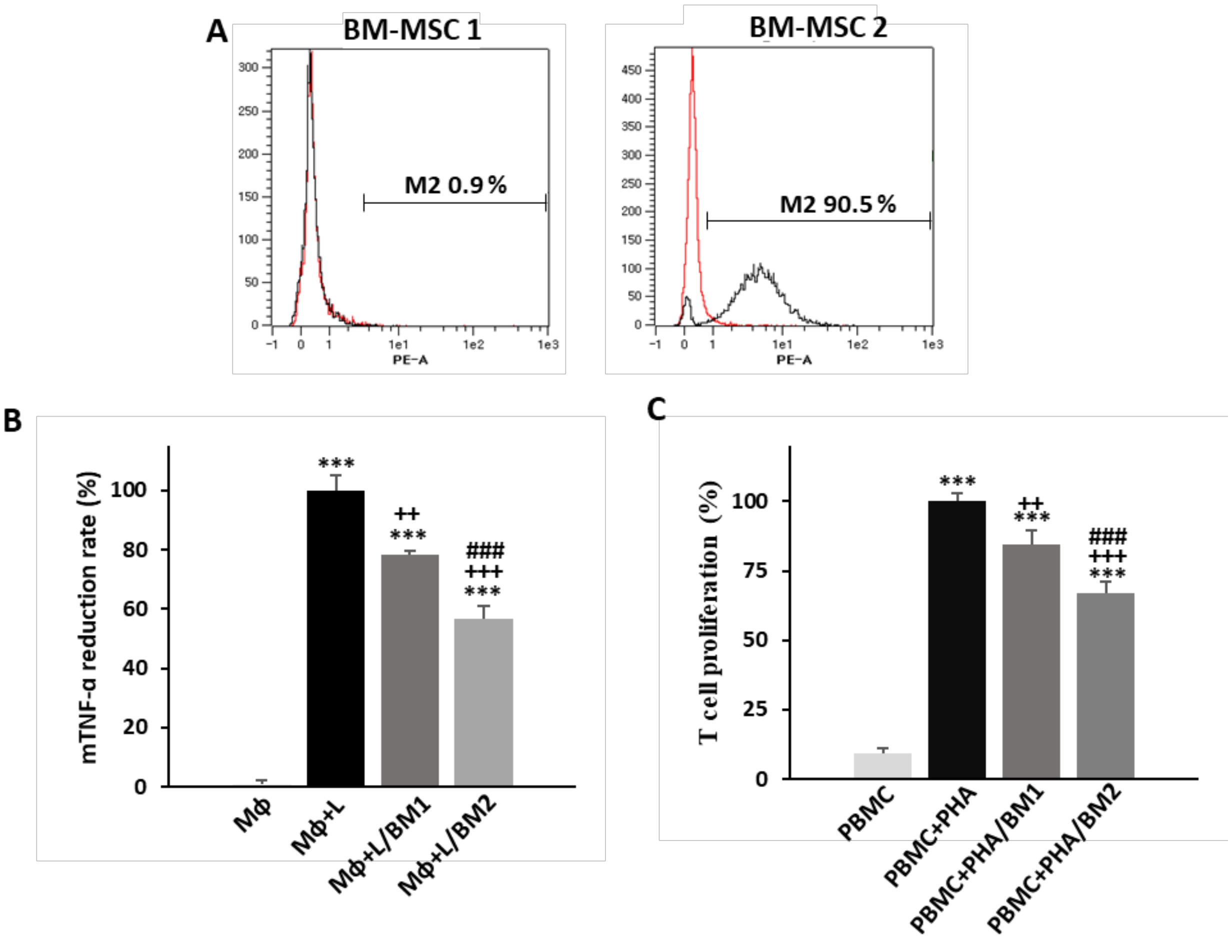

Figure S4. HLA-A2 expression in BM-MSCs from two different donors. (A) The protein expression levels of HLA-A2 were analyzed via flow cytometry. HLA-A2 expression was higher in BM1 compared to BM2 MSCs. (B) Raw 264.7 cells were exposed to LPS and co-cultured with BM-MSCs (1 or 2) for 2 days. Cell supernatants were analyzed for mouse TNF-*α* via ELISA. All MSCs exhibited anti-inflammatory effects. Two BM-MSCs exhibited significant difference in TNF-*α* suppression. Error bars represent means ± SD, n=5 per group; *** p <0.001, vs. MΦ, +++ p< 0.001, ++ p< 0.01 vs. MΦ+L, ### p< 0.01 vs. MΦ+L/BM1. (C) PBMCs were stimulated with PHA and were then co-cultured with MSCs for 3 days. The proliferation of T cells is shown as a percentage relative to the positive control (PBMC+PHA; set to 100%). All MSCs exhibited T cell-suppressive effects. Two groups showing significant differences in T cell proliferation. Error bars represent the means ± SD, n=5 per group; *** p <0.001, vs. PBMC, +++ p< 0.001, ++ p< 0.01 vs. PBMC+PHA, ### p< 0.01 vs. PBMC+PHA /BM1. MΦ; macrophage, L; LPS, PBMC; peripheral blood mononuclear cell, PHA; phytohemagglutinin.

Table S1. Basic information of UCB-MSCs.

UCB-MSCs	Surface marker		Differentiation
	Positive	Negative	
#1	Pass	Pass	Pass
#2	Pass	Pass	Pass
#3	Pass	Pass	Pass
#4	Pass	Pass	Pass
#5	Pass	Pass	Pass
#6	Pass	Pass	Pass
#7	Pass	Pass	Pass
#8	Pass	Pass	Pass
#9	Pass	Pass	Pass
#10	Pass	Pass	Pass
#11	Pass	Pass	Pass
#12	Pass	Pass	Pass
#13	Pass	Pass	Pass
#14	Pass	Pass	Pass
#15	Pass	Pass	Pass

UCBs were isolated from three independent donors (UCB #1–15). UCB-MSCs were characterized based on MSC surface marker expression and MSC differentiation potential (positive: CD73, CD90, CD105, and CD166 ≥ 80%; negative: CD14, CD45 ≤ 1.0%; differentiation: osteogenic, chondrogenic, adipogenic).

Table S2. Sequences of primers and siRNAs.

Construct	Sequence (5′-3′)
Scramble siRNA	UGGUUUACAUGUCGACUAA<br>UGGUUUACAUGUUGUGUGA<br>UGGUUUACAUGUUUUCUGA<br>UGGUUUACAUGUUUUCCUA
HLA-A2 siRNA	GAUGCUGAACAGUGACAAA<br>CGGAAAGCUUGCCUCAAUC<br>UUACAGUGUUUCUGGCUUA<br>GCUGGCGGAUCCAAGCAAA
HLA-A2 primer	ACUAAGAGUGGUCGAAGAA<br>GCACAGCAGCAGAUCGAUU

Table S3. Groups for the *in vivo* experiment

Group	Number	MSCs (cells)	PBS volume (μL)
Normal control	10	-	50
Hyperoxic lung injury	18	-	50
BPD+naïve MSC	15	1×10^5	50
BPD+con siRNA MSC	15	1×10^5	50
BPD+HLA-A2 siRNA MSC	15	1×10^5	50

Table S4. Surface marker expression among different stem cell populations

Transfection	CD14	CD45	HLA DR	CD73	CD90	CD105	CD166
Naive	-	-	-	+	+	+	+
Con siRNA	-	-	-	+	+	+	+
HLA-A2 siRNA	-	-	-	+	+	+	+

The cells were transfected under three conditions. Surface antigen expression was assessed via flow cytometry. Three condition cells were strongly positive for MSC-specific markers CD73, CD90, CD105, and CD166, while negative for CD14, CD45, and HLA-DR (+: more than 80%; -: less than 5%).
